# The Association of Four Common Polymorphisms from Four Candidate Genes (*COX-1*, *COX-2*, *ITGA2B*, *ITGA2*) with Aspirin Insensitivity: A Meta-Analysis

**DOI:** 10.1371/journal.pone.0078093

**Published:** 2013-11-14

**Authors:** Zhiyuan Weng, Xiaobo Li, Yuqiong Li, Jinxiu Lin, Feng Peng, Wenquan Niu

**Affiliations:** 1 Department of Cardiology, The First Affiliated Hospital of Fujian Medical University, Fuzhou, China; 2 State Key Laboratory of Medical Genomics, Rui Jin Hospital, School of Medicine, Shanghai Jiao Tong University, Shanghai, China; 3 Department of Hypertension, Rui Jin Hospital, School of Medicine, Shanghai Jiao Tong University, Shanghai, China; Sanjay Gandhi Medical Institute, India

## Abstract

**Objective:**

Evidence is mounting suggesting that a strong genetic component underlies aspirin insensitivity. To generate more information, we aimed to evaluate the association of four common polymorphisms (rs3842787, rs20417, rs201184269, rs1126643) from four candidate genes (*COX-1*, *COX-2*, *ITGA2B*, *ITGA2*) with aspirin insensitivity via a meta-analysis.

**Methods and Results:**

In total, there were 4 (353/595), 6 (344/698), 10 (588/878) and 7 (209/676) articles (patients/controls) qualified for rs3842787, rs20417, rs20118426 and rs1126643, respectively. The data were extracted in duplicate and analyzed by STATA software (Version 11.2). The risk estimate was expressed as odds ratio (OR) and 95% confidence interval (95% CI). Analyses of the full data set indicated significant associations of rs20417 (OR; 95% CI; P: 1.86; 1.44–2.41; <0.0005) and rs1126643 (2.37; 1.44–3.89; 0.001) with aspirin insensitivity under allelic model. In subgroup analyses, the risk estimate for rs1126643 was greatly potentiated among patients with aspirin semi-resistance relative to those with aspirin resistance, especially under dominant model (aspirin semi-resistance: 5.44; 1.42–20.83; 0.013 versus aspirin resistance: 1.96; 1.07–3.6; 0.03). Further grouping articles by ethnicity observed a stronger prediction of all, but rs20417, examined polymorphisms for aspirin insensitivity in Chinese than in Caucasians. Finally, meta-regression analyses observed that the differences in percentage of coronary artery disease (P = 0.034) and averaged platelet numbers (P = 0.012) between two groups explained a large part of heterogeneity for rs20417 and rs1126643, respectively.

**Conclusion:**

Our findings provide strong evidence that *COX-2* and *ITGA2* genetic defects might increase the risk of having aspirin insensitivity, especially for aspirin semi-resistance and in Chinese populations.

## Introduction

As a routine therapeutic agent, aspirin is prescribed widely for the prophylaxis of cardio-thrombotic events. The effect of aspirin is achieved by suppressing thromboxane production and further by inhibiting platelet activation and aggregation [1]. However, a considerable number of patients on aspirin therapy fail to reach this desired effect, and instead they experience major adverse vascular events, a phenomenon known as ‘aspirin insensitivity’ [2]. Since the discovery of this phenomenon, to unravel the underlying mechanisms of aspirin insensitivity so far remains a daunting task. Evidence is mounting suggesting that a strong genetic component underlies aspirin insensitivity [3], [4]. Literature, being abundant with candidate gene association studies [5]–[8], paves the way to determine how many genes and which genetic determinants are actually predisposing an individual to aspirin insensitivity [9]. However, the resultant associations are often not reproducible, likely due to the divergent ethnicity-specific genetic profiles, the population stratification and cryptic relatedness, the inadequate sample sizes, and the lack of adjustment for confounders [10]–[12].

To shed some light on this issue, we sought to evaluate the association of four common polymorphisms (rs3842787: 50C→T, rs20417: 765G→C, rs201184269: 1565T→C, rs1126643: 807C→T) with the risk of having aspirin insensitivity by conducting a meta-analysis of individual participant data from all qualified case-control studies. The four polymorphisms examined are mapped separately on four candidate genes: cyclooxygenase-1 (*COX-1*, chromosome 9q32-q33.3), cyclooxygenase-2 (*COX-2*, chromosome 1q25.2-q25.3), integrin, alpha 2b (*ITGA2B*, chromosome 17q21.32) and integrin alpha 2 (*ITGA2*, chromosome 5q11.2).

The selection of the four candidate genes is based on their pathogenic roles in platelet regulation. In brief, aspirin is reported to inhibit platelets by acetylating COX-1 and COX-2 enzymes, and further to block the production of thromboxane A2, a platelet agonist [1]. Especially, thromboxane A2, via transmitting intracellular signals into platelet, can activate the ITGA2B receptor, a platelet-membrane glycoprotein important for platelet aggregation [9]. ITGA2 serves as the platelet receptor of collagen that is a physiologically important activating agent of platelet aggregation [9]. Moreover, the selection of these four polymorphisms is based on the fact that if there are three or more independent studies investigating the same polymorphism in aforementioned four genes, data were synthesized accordingly.

## Methods

Meta-analysis of observational studies has particular challenges owing to the inherent biases and drawbacks in study design. We therefore carried out this meta-analysis according to the guidelines set forth by the Preferred Reporting Items for Systematic Reviews and Meta-analyses (PRISMA) statement [13] (See Checklist S1, PRISMA checklist).

### Search

PubMed, Wanfang (http://www.wanfangdata.com.cn) and China Biological Medicine (CBM) (http://sinomed.imicams.ac.cn/index.jsp) databases were searched for articles published in English or Chinese language before May 2013.

Eligibility of the retrieved articles was evaluated by reading the titles and the abstracts if necessary. Additional evaluation was extended by reviewing the bibliographies of articles and relevant reviews. The most compete and recent results were abstracted in case of multiple publications from the same study group. Articles with data on both aspirin resistance and semi-resistance were treated separately.

All qualified articles in the meta-analysis were approved by the ethics committee of each study, and written informed consents were obtained from all subjects before enrollment.

### Inclusion/exclusion criteria

Articles were included if (i) they evaluated the association of at least one of four polymorphisms (rs3842787, rs20417, rs201184269, rs1126643) with the risk of having aspirin insensitivity; (ii) they were conducted on a case-control or nested case-control study design; (iii) they provided the genotype and/or allele counts of examined polymorphisms between patients with aspirin insensitivity and controls in order to estimate odds ratio (OR) and 95% confidence interval (95% CI).

Articles were excluded if (i) they did not provide the genotype or allele counts of examined polymorphisms; (ii) they lacked either patient group or control group; (iii) they were experimental investigations or clinical trials; (iv) they were meeting abstracts, case reports/series, editorials, review articles, or non-English and non-Chinese publications.

### Data extraction

Data were extracted independently by two authors (Zhiyuan Weng and Wenquan Niu) on a standardized Excel template and were verified with disagreements settled by consensus.

From each article, information was extracted on the first author, publication year, ethnicity, type of aspirin insensitivity (aspirin resistance and aspirin semi-resistance), study design, the genotypes/alleles of examined polymorphisms, age, gender, body mass index (BMI), smoking, triglyceride, total cholesterol (TC), high-density lipoprotein cholesterol (HDLC), low-density lipoprotein cholesterol (LDLC), platelet number, as well as the percentages of hypertension, diabetes, dyslipidemia, coronary artery disease (CAD), cerebrovascular disease (CVD).

### Statistical analysis

Data management and statistical analyses were conducted using STATA software version 11.2 (Stata Corp LP, College Station, TX, USA) for Windows. Risk estimate was expressed as OR with 95% CI. Hardy-Weinberg equilibrium was tested by χ^2^ test or Fisher's exact test if necessary.

The random-effects model using the DerSimonian and Laird method was adopted. Statistical heterogeneity was assessed by χ^2^ test and was quantified using the *I* ^2^ statistic (ranging from 0 to 100%), which is defined as the percentage of the observed between-study variability that is due to heterogeneity rather than chance. Met-regression analyses were conducted to estimate the potential confounding of risk factors such as age and gender.

Publication bias was assessed using the Egger regression test. The Egger's test detects Begg's funnel plot asymmetry by determining whether the intercept deviates significantly from zero in a regression of the standardized effect estimates against their precision. Significance was judged at P<0.05 except for the *I* ^2^ statistic and Egger's test at P<0.1.

## Results

### Qualified articles

The initial search retrieved 154 potentially relevant references (118 published in English and 36 in Chinese). Applying our inclusion/exclusion criteria left 21 qualified articles [5]–[8], [14]–[30], in which the association of four examined polymorphisms with aspirin insensitivity was examined.

**Figure 1:**
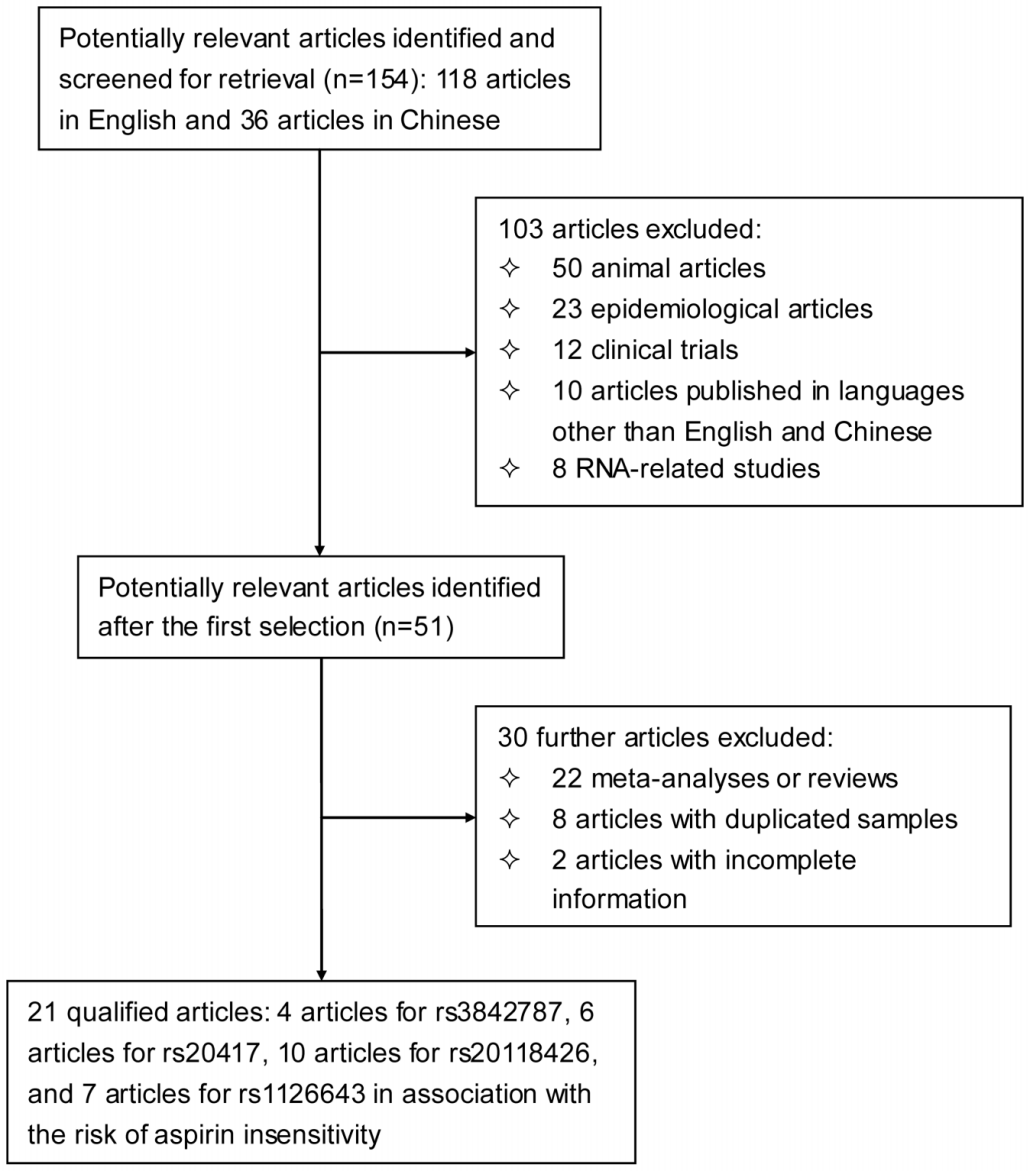
Flow diagram of search strategy and study selection.

A flow diagram schematizing the selection process of identified articles with specific reasons, and the baseline characteristics of all qualified articles are presented in Figure 1 and Tables 1 and 2, respectively. The retrieved articles were published between 2003 and 2012, and 11 of them were written in Chinese and 10 in English. There were a total of 4 [6], [16], [17], [29], 6 [14], [17], [21], [24], [25], [29], 10 [5], [7], [8], [15], [18], [19], [26]–[28], [30] and 7 [8], [19], [22], [23], [28]–[30] qualified articles and 353/595, 344/698, 588/878 and 209/676 cases/controls for rs3842787, rs20417, rs20118426 and rs1126643, respectively. Five articles that reported both aspirin resistance and semi-resistance were treated separately [16], [19]–[21], [23]. Therefore, there were 26 comparisons in final analysis.

**Table 1:** The baseline characteristics of all qualified articles.

Author (year)	Ethnicity	Age, years		Gender (Males, %)		BMI, kg/m^2^		Smoking (%)		Hypertension (%)		Diabetes (%)		Dyslipidemia (%)	
		Cases	Conts	Cases	Conts	Cases	Conts	Cases	Conts	Cases	Conts	Cases	Conts	Cases	Conts
**Macchi L (2003)**	Caucasian	68.5	64.7	62.07	85.51	NA	NA	24.14	13.04	55.17	50.72	20.69	14.49	75.86	71.01
**Papp E (2005)**	Caucasian	66	65	57.98	57.83	NA	NA	35.29	31.93	92.44	82.53	21.85	22.89	59.66	66.27
**Pamukcu B (2005)**	Mixed	59.6	55.5	81.4	76.4	NA	NA	62.79	62.73	60.47	62.73	16.28	18.63	NA	NA
**Gonzalez CR (2005)**	Caucasian	35.6	NA	45.83	NA	NA	NA	NA	NA	0	0	NA	NA	NA	NA
**Fontana P (2006)**	Caucasian	29.3	27.5	100	100	23.5	23.2	0	0	NA	NA	NA	NA	NA	NA
**Bernardo E (2006)**	Caucasian	65	61	20	76.47	NA	NA	8	9.8	60	47.06	56	31.37	48	52.94
**Su G (2007) a**	Chinese	68.7	64.6	55.6	85.3	NA	NA	33.3	11.3	55.6	51.3	22.2	15.3	NA	NA
**Su G (2007) b**	Chinese	65.1	64.6	65.9	85.3	NA	NA	31.7	11.3	53.7	51.3	19.5	15.3	NA	NA
**Wu W (2007)**	Chinese	62.6	61.79	42.99	55.52	NA	NA	NA	NA	37.38	23.97	38.32	16.09	NA	NA
**Lev E (2007)**	Mixed	67.2	65.3	41.67	71.3	30.9	29.8	33.33	32.41	83.33	84.26	NA	NA	83.33	70.37
**Kranzhofer R (2007)**	Caucasian	NA	NA	NA	NA	NA	NA	NA	NA	NA	NA	NA	NA	NA	NA
**Zhang J (2009) a**	Chinese	76.2	73.1	60.87	70.83	NA	NA	NA	NA	NA	NA	NA	NA	NA	NA
**Zhang J (2009) b**	Chinese	75.5	73.1	36.73	70.83	NA	NA	NA	NA	NA	NA	NA	NA	NA	NA
**Xin X (2009)**	Chinese	60.1	58.6	22.2	60.4	NA	NA	NA	NA	NA	NA	NA	NA	NA	NA
**Kunicki TJ (2009)**	Caucasian	72.6	70.9	49.12	56.16	NA	NA	23.68	18.34	55.26	53.01	9.65	14.33	NA	NA
**Zhou Q (2010) a**	Chinese	73.28	65.68	33.33	68.92	NA	NA	20.51	27.03	87.18	81.08	35.9	16.22	61.54	18.92
**Zhou Q (2010) b**	Chinese	70.3	65.68	38.46	68.92	NA	NA	38.46	27.03	92.31	81.08	61.54	16.22	76.92	18.92
**Li A (2010)**	Chinese	62	NA	59.09	NA	NA	NA	NA	NA	NA	NA	NA	NA	NA	NA
**Zhao Y (2011)**	Chinese	73.73	72.41	60.61	60.27	27.04	26.5	24.24	19.18	84.85	78.08	57.6	63	39.39	34.25
**Mao X (2011)**	Chinese	72.68	73.79	61.29	55.17	NA	NA	NA	NA	90.32	79.31	32.26	17.24	NA	NA
**Li C (2011) a**	Chinese	61.03	NA	37.84	58.26	NA	NA	37.5	26.96	37.5	18.26	50	25.22	NA	NA
**Li C (2011) b**	Chinese	61.03	NA	37.93	58.26	NA	NA	27.59	26.96	31.03	18.26	48.28	25.22	NA	NA
**Wang Y (2012)**	Chinese	68.5	19.18	72.22	79.63	NA	NA	NA	NA	82.4	60.2	31.4	29.6	37.3	34.3
**Wang B (2012)**	Chinese	75.75	76.79	65.6	64.84	23.79	23.69	0	0	45.2	39.56	NA	NA	NA	NA
**Li X (2012) a**	Chinese	76.33	73.88	69.44	66.23	24.93	25.29	30.56	22.08	75	71.43	52.78	45.89	38.89	39.39
**Li X (2012) b**	Chinese	74.02	73.88	64.02	66.23	24.93	25.29	25	22.08	66.46	71.43	42.07	45.89	35.37	39.39

*Abbreviations:* Conts, controls; BMI, body mass index; NA, data not available.

**Table 2:** The baseline characteristics of the study populations.

Author (year)	CAD (%)		CVD (%)		TC, mmol/L		TG, mmol/L		LDLC, mmol/L		HDLC, mmol/L		Platelet number	
	Cases	Conts	Cases	Conts	Cases	Conts	Cases	Conts	Cases	Conts	Cases	Conts	Cases	Conts
**Macchi L (2003)**	NA	NA	NA	NA	NA	NA	NA	NA	NA	NA	NA	NA	296	275
**Papp E (2005)**	55.44	55.44	24.21	24.21	NA	NA	NA	NA	NA	NA	NA	NA	NA	NA
**Pamukcu B (2005)**	NA	NA	NA	NA	4.68	4.76	1.7	1.7	1.01	1.03	2.79	2.84	230.58	221.53
**Gonzalez CR (2005)**	NA	NA	NA	NA	NA	NA	NA	NA	NA	NA	NA	NA	244	256
**Fontana P (2006)**	NA	NA	NA	NA	NA	NA	NA	NA	NA	NA	NA	NA	214.9	213.9
**Bernardo E (2006)**	100	100	NA	NA	NA	NA	NA	NA	NA	NA	NA	NA	NA	NA
**Su G (2007) a**	NA	NA	NA	NA	NA	NA	NA	NA	NA	NA	NA	NA	276	265
**Su G (2007) b**	NA	NA	NA	NA	NA	NA	NA	NA	NA	NA	NA	NA	237	265
**Wu W (2007)**	NA	NA	NA	NA	5.03	4.96	1.81	1.7	1.25	1.29	3.15	2.86	NA	NA
**Lev E (2007)**	NA	NA	NA	NA	NA	NA	NA	NA	NA	NA	NA	NA	236.9	204.8
**Kranzhofer R (2007)**	100	100	NA	NA	NA	NA	NA	NA	NA	NA	NA	NA	NA	NA
**Zhang J (2009) a**	100	100	NA	NA	NA	NA	NA	NA	NA	NA	NA	NA	NA	NA
**Zhang J (2009) b**	100	100	NA	NA	NA	NA	NA	NA	NA	NA	NA	NA	NA	NA
**Xin X (2009)**	66.06	NA	33.94	3	NA	NA	NA	NA	NA	NA	NA	NA	230	228
**Kunicki TJ (2009)**	24.56	23.5	71.93	73.35	NA	NA	NA	NA	NA	NA	NA	NA	202.7	187.4
**Zhou Q (2010) a**	NA	NA	NA	NA	NA	NA	NA	NA	NA	NA	NA	NA	196.51	195.05
**Zhou Q (2010) b**	NA	NA	NA	NA	NA	NA	NA	NA	NA	NA	NA	NA	202.5	195.05
**Li A (2010)**	100	100	NA	NA	NA	NA	NA	NA	NA	NA	NA	NA	NA	NA
**Zhao Y (2011)**	NA	NA	NA	NA	5.78	5.62	1.7	1.68	1.34	1.39	3.02	3.07	NA	NA
**Mao X (2011)**	NA	NA	54.84	62.07	4.81	4.47	1.56	1.45	1.05	1.04	2.98	2.75	211.65	193.57
**Li C (2011) a**	NA	NA	NA	NA	5.42	5.2	NA	NA	NA	NA	3.14	3.46	237	241
**Li C (2011) b**	NA	NA	NA	NA	5.07	5.2	NA	NA	NA	NA	3.35	3.46	227	241
**Wang Y (2012)**	11.76	20.37	50.98	45.37	4.64	4.44	1.54	1.72	1.28	1.21	2.76	2.61	NA	NA
**Wang B (2012)**	42.4	37.91	NA	NA	NA	NA	NA	NA	NA	NA	NA	NA	NA	NA
**Li X (2012) a**	66.67	56.71	44.44	36.8	4.75	5.18	1.78	1.57	1.28	1.34	2.82	2.3	207.6	207.81
**Li X (2012) b**	55.49	56.71	35.37	36.8	4.82	5.18	1.63	1.57	1.27	1.34	2.85	2.3	209.3	207.81

*Abbreviations:* Conts, controls; CAD, coronary artery disease; CVD, cerebrovascular disease; TC, total cholesterol; TG, triglyceride; LDLC, low-density lipoprotein cholesterol; HDLC, high-density lipoprotein cholesterol; NA, data not available.

### Study characteristics

17 of 26 comparisons involved Chinese subjects (12 from north China and 5 from south China), 7 involved Caucasians, and 2 involved the mixed populations. Deviations from Hardy-Weinberg equilibrium were observed for rs20118426 [26], [28] and rs1126643 [23] in 2 comparisons, respectively.

The risk-allele frequencies of rs3842787, rs20417, rs20118426 and rs1126643 were respectively 3.93%, 20.36%, 11.82% and 50.67% in patients, and 4.07%, 12.16%, 11.71% and 30.4% in controls. By ethnicity, the risk-allele frequencies of rs3842787, rs20417, rs20118426 and rs1126643 were respectively 11.64%, 25.0%, 16.09% and 40.77% in Caucasian patients and 11.99%, 9.38%, 17.41% and 32.55% in Caucasian controls, and the corresponding frequencies were respectively 0.08%, 19.59%, 6.17% and 58.58% in Chinese patients and 10.82%, 12.62%, 3.88% and 28.69% in Chinese controls.

### Overall analyses

Taking all available comparisons together for each polymorphism observed significant association of *COX-2* gene rs20417 and *ITGA2* gene rs1126643 with aspirin insensitivity, whereas no significance was found for *COX-1* gene rs3842787 and *ITGA2B* gene rs201184269 under both allelic and dominant models (Table 3). For instance, risk estimates conferred by rs1126643-T allele reached as high as 2.37 (95% CI: 1.44–3.89; P = 0.001) for the occurrence of aspirin insensitivity relative to the alternative allele, and this estimation was more prominent under dominant model (OR = 2.81; 95% CI: 1.54–5.13; P = 0.001), despite marked between-study heterogeneity (P<0.01 for *I* ^2^) and low probability of publication bias as reflected by Egger's test (P>0.2). It is also worth mentioning that the significant association of rs20417 with aspirin insensitivity was immune from the disturbance of heterogeneity and publication bias. In addition, excluding comparisons with genotypes deviating from Hardy-Weinberg equilibrium yielded almost similar results (Table 3).

**Table 3:** Overall and subgroup analyses of four examined polymorphism with aspirin insensitivity under both allelic and dominant models.

Polymorphisms	Group or subgroups	Allelic model			Dominant model		
		OR; 95% CI; P	*I* ^2^; P_χ2_	P_Egger_	OR; 95% CI; P	*I* ^2^; P_χ2_	P_Egger_
**rs3842787**	Overall	1.19; 0.77–1.83; 0.424	0.0%; 0.939	0.665	1.25; 0.75–2.08; 0.397	0.0%; 0.934	0.616
**(50C→T)**	AR	1.19; 0.77–1.84; 0.441	0.0%; 0.823	NA	1.24; 0.74–2.09; 0.415	0.0%; 0.81	NA
	Chinese	1.69; 0.21–13.75; 0.626	0.0%; 0.852	NA	1.68; 0.2–13.77; 0.629	0.0%; 0.855	NA
	Caucasians	1.17; 0.76–1.82; 0.475	0.0%; 0.61	NA	1.22; 0.72–2.07; 0.452	0.0%; 0.575	NA
**rs20417** *	Overall	1.86; 1.44–2.41; <0.0005	0.0%; 0.7	0.521	1.9; 1.4–2.58; <0.0005	0.0%; 0.59	0.495
**(765G→C)**	Chinese	1.84; 1.42–2.38; <0.0005	0.0%; 0.64	0.846	1.86; 1.36–2.53; <0.0005	0.0%; 0.567	0.937
**rs20118426**	Overall	1.12; 0.71–1.76; 0.64	50.2%; 0.034	0.406	1.06; 0.64–1.76; 0.826	48.7%; 0.041	0.221
**(1565T→C)**	HWE	1.17; 0.73–1.89; 0.51	27.8%; 0.206	0.317	1.2; 0.67–2.15; 0.534	33.2%; 0.163	0.769
	AR	1.07; 0.69–1.68; 0.771	50.4%; 0.04	0.046	1.0; 0.61–1.64; 0.994	48.1%; 0.052	0.221
	Chinese	4.07; 0.67–24.81; 0.128	50.0%; 0.136	NA	4.19; 0.71–24.65; 0.113	45.3%; 0.161	NA
	Caucasians	0.98; 0.54–1.77; 0.934	61.5%; 0.034	0.062	0.88; 0.46–1.68; 0.702	57.5%; 0.052	0.204
**rs1126643**	Overall	2.37; 1.44–3.89; 0.001	76.1%; <0.0005	0.468	2.81; 1.54–5.13; 0.001	54.7%; 0.024	0.207
**(807C→T)**	HWE	2.32; 1.23–4.39; 0.009	80.5%; <0.0005	0.759	3.12; 1.34–7.24; 0.008	66.2%; 0.007	0.246
	AR	1.85; 1.11–3.07; 0.017	54.5%; 0.052	0.07	1.96; 1.07–3.6; 0.03	28.5%; 0.221	0.012
	ASR	3.35; 1.28–8.77; 0.014	87.8%; <0.0005	0.292	5.44; 1.42–20.83; 0.013	73.2%; 0.024	NA
	Chinese	3.58; 1.86–6.92; <0.0005	76.5%; 0.002	0.407	4.98; 2.07–12.01; <0.0005	51.0%; 0.086	0.292
	Caucasians	1.29; 0.89–1.85; 0.176	0.0%; 0.418	0.374	1.49; 0.86–2.55; 0.152	3.4%; 0.376	0.21

*Abbreviations:* OR, odds ratio; 95% CI, 95% confidence interval; AR, aspirin resistance; ASR, aspirin semi-resistance; HWE, Hardy-Weinberg equilibrium.

* For rs20417, all qualified articles did not report comparisons with aspirin semi-resistance.

### Subgroup analyses

To estimate the influence of categorical confounders, separate analyses were performed within strata involving two or more comparisons (Table 3). By type of aspirin insensitivity, data were insufficient for rs3842787, rs20417 and rs20118426 to assess their associations with aspirin semi-resistance. For rs1126643, risk estimates was remarkably potentiated among patients with aspirin semi-resistance compared with those with aspirin resistance, especially under dominant model (aspirin semi-resistance: OR = 5.44; 95% CI: 1.42–20.83; P = 0.013 versus aspirin resistance: OR = 1.96; 95% CI: 1.07–3.6; P = 0.03). Heterogeneity was improved greatly for aspirin resistance comparisons.

Further grouping articles by ethnicity of study populations (mainly Chinese and Caucasian) observed the enhanced prediction of all examined polymorphisms except for rs20417 in Chinese compared with Caucasians (Table 3). Take rs1126643 for example, the odds of aspirin insensitivity in Chinese was nearly threefold relative to in Caucasians under both allelic (OR: 3.58 versus 1.29) and dominant (OR: 4.98 versus 1.49) models. However, a note of caution should be added because heterogeneity might potentially restrict the interpretation of risk estimates in Chinese (allelic model: *I* ^2^ = 76.5% and dominant model: *I* ^2^ = 51.0%).

### Meta-regression analyses

To further explore other potential sources of heterogeneity, a multivariable meta-regression model incorporating available study-level continuous covariates was conducted. Differences in percentage of CAD between patients and controls explained a large part of heterogeneity for rs20417 (P = 0.034). Moreover, averaged platelet number was a significant source of heterogeneity for rs1126643 (P = 0.012).

## Discussion

The most noteworthy finding of this meta-analysis was that *COX-2* and *ITGA2* genetic defects might increase the risk of having aspirin insensitivity, especially for aspirin semi-resistance and in Chinese populations. However, these significant associations were resulted from pooling a small number of studies with limited sample sizes, and therefore our findings must be interpreted with caution.

Aspirin insensitivity is a poorly characterized phenomenon in both clinical and laboratory contexts. Although the laboratory diagnosis of aspirin insensitivity cannot substitute clinical diagnosis, there is every reason to believe that most if not all laboratory assays do reflect some rationale and degree of validity and sensitivity, albeit variable, of such insensitivity [31]. If not, any real aspirin insensitive impact on clinical outcomes would be undetectable. A previous meta-analysis by the Antithrombotic Trialists' Collaboration documented that oral antiplatelet drugs in secondary prevention decreased the risk of a subsequent myocardial infarction by 25% and mortality by 20% among patients at high risk for cardiovascular events [32]. However, even usage of such drugs also led to a residual rate of re-hospitalization among about 15% of patients with diagnosed ischemic heart disease [33]. One possible reason for this high readmission rate might be that there is a genetic component in the inherited susceptibility to aspirin insensitivity. As the number of candidate gene association studies is rapidly growing, one practical way to unveil the genetic basis of aspirin insensitivity is to systematically pool available data to obtain robust, replicable findings.

In this study, we evaluated the association of four common polymorphisms from four logical candidate genes (*COX-1*, *COX-2*, *ITGA2B*, *ITGA2*) with aspirin insensitivity via a meta-analysis. Our overall findings demonstrated the contributory roles of *COX-2* and *ITGA2* genetic polymorphisms in susceptibility to aspirin insensitivity; however, after stratifying studies by ethnicity, the risk estimates were strongly reinforced in populations of Chinese origin, relative to that of Caucasian origin. One possible explanation for this divergence is genetic heterogeneity across races and ethnicities. For example, the average frequency of *ITGA2* gene rs1126643-T allele was 40.77% in Caucasian patients with aspirin insensitivity, but was as exceedingly high as 58.58% in Chinese patients. It is not uncommon to encounter genetic heterogeneity in any disease identification strategy. This ethnicity-specific effect suggests that different genetic backgrounds may account for this discrepancy or that different populations may have different linkage disequilibrium patterns due to the evolutionary history. Usually, a locus is in close linkage with another nearby causal locus in one ethnic group but not in another [34]. As a consequence, there is a need to construct a database of aspirin insensitivity-susceptibility genes or polymorphisms in each racial/ethnic group.

To further account for other potential sources of heterogeneity, we employed a multivariable meta-regression model by incorporating several study-level covariates besides subgroup analyses. Interestingly, differences in percentage of CAD between patients and controls set out to be a potential source of heterogeneity across studies for *COX-2* gene rs20417, suggesting its regulatory role in cardiovascular system [35], [36]. Moreover, the averaged platelet numbers also explained a large part of heterogeneity for the relevance of *ITGA2* gene rs1126643 to aspirin insensitivity, which further strengthened our overall findings. However, it should be emphasized that meta-regression, although enabling consideration of various covariates, does not have the methodological robustness of a properly designed study that is intended to test the effect of these covariates formally [37]. On the other hand, because meta-regression analysis involved studies of limited sample size, it might be underpowered to detect a small or moderate effect. Although statistical biases could not be ruled out and an indication of heterogeneity was noted for some comparisons, there was no evidence of publication bias in this meta-analysis as reflected by Egger's test, indicating the robustness of our findings.

Interpretation of this study, however, should consider several limitations. First, although our statistical tests showed low probability of publication bias, potential selection bias cannot be excluded, because we only retrieved articles published in English or Chinese language [38]. Second, although a set of subgroup analyses had been undertaken, significant heterogeneity still persisted in some subgroups, limiting the interpretation of pooled risk estimates. Moreover for some polymorphisms, given the relatively small sample sizes, especially in subgroups, more large, well-designed studies are warranted to quantify risk estimates reliably. Third, we only involved four polymorphisms from four candidate genes in biological susceptibility to aspirin insensitivity. It is likely that the potential susceptibility of these polymorphisms to aspirin insensitivity is diluted or masked by gene-gene or gene-environment interactions. Therefore, the jury must refrain from drawing a final conclusion until large, well-designed, prospective studies confirm or refute our findings.

Despite these limitations, our findings demonstrated that the *COX-2* and *ITGA2* genetic defects might increase the risk of having aspirin insensitivity, especially for aspirin semi-resistance and in Chinese populations. Our findings also leave open the question of divergent genetic profiles across ethnic groups. Nonetheless, this meta-analysis provides supporting evidence for further investigation on the pathophysiological mechanisms of *COX-2* and *ITGA2* genes in the development of aspirin insensitivity.

## Supporting Information

Checklist S1 **PRISMA Checklist.**
(DOC)
